# m6AConquer: a consistently quantified and orthogonally validated database for the *N*^6^-methyladenosine (m^6^A) epitranscriptome

**DOI:** 10.1093/nar/gkaf1204

**Published:** 2025-12-03

**Authors:** Xichen Zhao, Haokai Ye, Dan He, Hongxi Liu, Tenglong Li, Daniel J Rigden, Zhen Wei

**Affiliations:** Department of Biosciences and Bioinformatics, Xi’an Jiaotong-Liverpool University, Suzhou 215123, China; Institute of Systems, Molecular and Integrative Biology, University of Liverpool, Liverpool L69 7ZB, UK; Department of Biosciences and Bioinformatics, Xi’an Jiaotong-Liverpool University, Suzhou 215123, China; Institute of Systems, Molecular and Integrative Biology, University of Liverpool, Liverpool L69 7ZB, UK; Department of Biosciences and Bioinformatics, Xi’an Jiaotong-Liverpool University, Suzhou 215123, China; Department of Biosciences and Bioinformatics, Xi’an Jiaotong-Liverpool University, Suzhou 215123, China; Wisdom Lake Academy of Pharmacy, Xi’an Jiaotong-Liverpool University, Suzhou 215123, China; Institute of Systems, Molecular and Integrative Biology, University of Liverpool, Liverpool L69 7ZB, UK; Department of Biosciences and Bioinformatics, Xi’an Jiaotong-Liverpool University, Suzhou 215123, China; Institute of Life Course and Medical Sciences, University of Liverpool, Liverpool L7 8TX, UK

## Abstract

The proper placement of *N*^6^-methyladenosine (m^6^A) on mRNA is essential for normal cell function, and its disruption is linked to numerous human diseases. The rapid growth of m^6^A data from diverse sequencing technologies presents challenges for integrative analysis due to technique-specific biases and inconsistent processing. While existing databases provide valuable catalogs, their reliance on aggregating pre-processed results can propagate inconsistencies. To overcome these limitations, we present m6AConquer, a database founded on reproducible quantification. We systematically re-processed raw data from 10 distinct profiling methods, including high-resolution GLORI and eTAM-seq, quantifying methylation at millions of consensus sites across human and mouse. Our rigorous pipeline features uniform site-calling and false-positive calibration with in vitro transcribed (IVT) controls where available. By leveraging a reproducibility-based framework across technically orthogonal methods, we identified over 135300 orthogonally validated m^6^A sites in human (IDR < 0.05). Beyond this validated methylome, m6AConquer provides matched multi-omics data (gene expression, splicing, variants) and identifies m^6^A quantitative trait loci (m^6^A QTLs) to link RNA modification to genetic regulation and disease. Offering intuitive query tools, interactive visualizations, and downloadable, analysis-ready data matrices, m6AConquer provides a standardized resource for rigorous exploration of the roles of m^6^A in biology and medicine, freely accessible at https://rnamd.org/m6aconquer/.

## Graphical Abstract

**Graphical Abstract:**
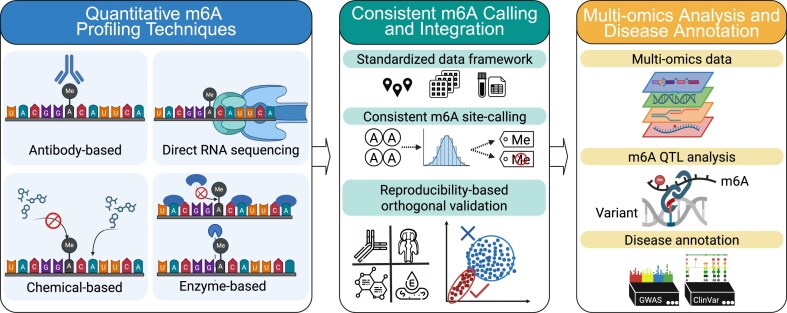

## Introduction

*N* ^6^-methyladenosine (m^6^A) modification is one of the most widely studied RNA chemical modifications in eukaryotic cells [1]. m^6^A epitranscriptomically regulates gene expression via specifying RNA half-life [2–8]. It is the most abundant type of RNA modification, topologically and functionally similar to DNA 5mC modification [9]. The basal constitutional level of m^6^A on transcripts functions as a negative gene expression regulation mechanism by targeting the modified RNA to P-bodies for degradation [1, 4, 5, 9, 10]. The installation of m^6^A on transcripts is mainly dictated by the DRACH consensus motif, but it is suppressed in regions close to splicing junctions [11]. This mechanism makes the intron-exon architecture a key determinant of RNA half-life, with longer exons harboring more m^6^A modifications [12–14].

m^6^A modification state is dynamically regulated by protein factors, including writers (*METTL3, METTL14, WTAP, KIAA1429*), erasers (*FTO, ALKBH5*), and readers (*YTHDF1-3, YTHDC1-2, HNRNPC*) [6, 15–23]. The specific m^6^A regulators allow the same m^6^A site link to distinct downstream outcomes via alternative mechanisms. m^6^A can also interact with epigenetic modules, including histone methylation (H3K36me3) and transposable element (SINEs and LINEs) expression [24–30]. m^6^A on noncoding RNAs, such as miRNAs and carRNAs, has also been reported to influence their processing and function. Furthermore, m^6^A plays a crucial role in fundamental cellular processes such as the cell cycle, differentiation, and stress responses [4, 31–36]. Accordingly, m^6^A is known to be involved in the development of disease and other medical conditions, including acute myelogenous leukemia (AML) [37–39], depression [40–42], and multiple cancers [43–46]. Perturbation of m^6^A regulators, like *FTO*, can also impact crop yields, as observed in *maize* [47–49].

Existing sequencing techniques for profiling the m^6^A methylome fall into four main groups: antibody-assisted, enzyme-assisted, chemical-assisted, and direct RNA sequencing methods. Antibody-based approaches, such as MeRIP-seq [15, 50] and miCLIP [51], enrich methylated RNA fragments through immunoprecipitation. Enzyme-assisted methods like eTAM-seq [52] and MAZTER-seq [53] use site-specific enzymatic cleavage or base-conversion reactions. Chemical-assisted protocols, including GLORI [54], convert unmethylated adenosines to inosines, facilitating quantitative discrimination. Lastly, direct RNA sequencing leverages distinct ionic current patterns generated when m^6^A-modified nucleotides pass through nanopores [55–58].

Despite their contributions to transcriptome-wide m^6^A mapping, each method has specific limitations. Antibody-based techniques frequently exhibit high false-positive rates (up to ∼30%), mainly because the antibody binds unmodified RNA regions whose sequence motifs resemble those typically found near true m^6^A-modified sites [59]. The high coverage of these regions in highly expressed transcripts further increases the likelihood of false-positive calls [59, 60]. Enzyme-assisted approaches often experience incomplete or inaccurate enzymatic reactions, exemplified by false-positive rates exceeding 50% in MAZTER-seq [59, 60]. Direct RNA sequencing continues to face challenges from limited IVT-generated training data and overlapping current signatures, complicating accurate site detection [59, 61]. Similarly, chemical-assisted methods like GLORI can encounter issues with incomplete conversions influenced by RNA secondary structures [62, 63].

Consequently, most existing methods lack reliable absolute quantification and exhibit inherent limitations in site resolution, making their measurements imprecise and requiring orthogonal validation to accurately characterize the transcriptome-wide distribution and dynamics of m^6^A methylation. In recent studies, technically independent validations have been increasingly adopted, where intersections between techniques reinforce the understanding of the true m^6^A installation mechanism in the transcriptome [12, 60, 64, 65]. For example, using common sites from miCLIP, m6A- SAC-seq, DART-seq, and GLORI, Anna *et al.* show that m^6^A formation is driven by the splice-site exclusion mechanism [12, 13]. Another study using a similar integrative analysis found that m^6^A does not promote protein translation [65].

While several valuable databases, including m6A-Atlas2, MeT-DB, RMBase2, REPIC, DirectRMDB, and Sci-ModoM, have emerged to catalogue m^6^A sites [66–71], the field still grapples with a fundamental challenge: the lack of a standardized and validated data-sharing framework for integrative analysis. Current databases typically act as data aggregators, compiling pre-computed peaks or modification sites from individual publications. This approach, however, inherits and propagates critical limitations. Firstly, it results in inconsistent site reporting, as different studies employ different peak-calling algorithms and statistical thresholds, making direct comparisons across datasets unreliable [59]. Secondly, these databases almost exclusively report “positive” sites, neglecting the vast landscape of high-coverage, low-methylated or unmethylated adenosine loci, which are essential for building generalizable predictive models and understanding regulatory specificity. This focus on pre-identified positive sites, common in many current resources, leads to a biased view of the methylome.

To address these critical challenges, we introduce a reproducibility-based integration strategy that moves beyond conventional data aggregation. Here, we present m6AConquer, a resource built upon this principle, which introduces three key advances:

**Consistent quantification**: We established a unified quantification strategy by processing all raw data from scratch. Instead of aggregating disparate published peak lists, we systematically re-quantified data from ten distinct profiling technologies against a unified set of over 5.5 million standardized loci across human and mouse. This process ensures two key advantages: first, every sample is measured against the same set of loci, enabling direct and reliable comparisons; second, it generates complete, matrix-based quantifications for all sites, eliminating the missing values inherent in peak-only databases. This homogeneous quantification is further enhanced by a uniform site-calling procedure and empirical false-positive calibration using in vitro transcribed (IVT) controls.
**Orthogonal validation**: A core improvement of our database is an integration strategy based on the Irreproducible Discovery Rate (IDR), a well-established standard in ENCODE projects [72]. We adapted this statistical framework to assess the quantitative agreement of methylation level (m^6^A ratio) between technically orthogonal methods, i.e. those rooted in distinct biochemical principles (e.g. antibody enrichment versus enzyme-based conversion). By demanding reproducibility across independent techniques, this approach rigorously filters out technology-specific artifacts and moves beyond simple site overlaps, resulting in a reliable set of orthogonally validated m^6^A sites.
**Multi-omics integration**: Finally, m6AConquer is the first resource in this field to provide matched, sample-specific multi-omics data, including transcriptomics, alternative splicing, and genetic variants, within a unified and analysis-ready format. This rich integration facilitates direct exploration of m^6^A’s functional consequences. As a demonstration, we used the multi-omics quantification to identify m^6^A quantitative trait loci (m^6^A QTLs) on our well-supported set of orthogonally validated sites. These associations can link genetic variants to RNA modification levels and potential disease risk. The QTL-based approach complements existing databases that rely on in-silico mutation effect predictions, which have shown inconsistencies with empirical m^6^A QTL evidence [73]. Key features distinguishing m6AConquer from other databases are summarized in Table 1.

**Table 1.** Comparison of features across M6aconquer, m6a-atlas, m6a-atlas2, Direct-RMDB, and Sci-ModoM about m^6^A modification on the human genome

Item	m6A-Conquer	m6A-Atlas	m6A-Atlas2	Direct-RMDB	Sci-ModoM
Quantification techniques	10	1	1	1	5
m^6^A reference sites	2 788 705	178 049	427 760	195 871	894 098
Samples/cells	1393	920	1481	18	80
Unified site-calling and quantification	✓	–	–	–	–
Orthogonal reproducible integration	✓	–	–	–	–
Standardized matrix datatype	✓	–	–	–	–
Transcriptomics and alternative splicing	✓	✓	✓	✓	–
Sample-specific SNP data	✓	–	–	–	–
m^6^A QTL	✓	–	–	–	–

m6AConquer is therefore presented not merely as a database, but as an inclusive data discovery platform. It provides the community with consistently quantified m^6^A signals and a stringently defined set of orthogonally validated sites, all organized within analysis-ready matrix-based data structures. Through an intuitive web interface, users can query, visualize, and download these large-scale and diverse datasets, facilitating reliable and detailed investigation into the mechanisms and functions of RNA methylation. All data, tools, and documentation are freely available at https://rnamd.org/m6aconquer/.

## Materials and methods

### Data collection and preprocessing

Raw sequencing data were collected from 10 quantitative m^6^A profiling technologies, spanning antibody-assisted (MeRIP-seq [66], m6ACE-seq [74, 75]), enzyme-assisted (eTAM-seq [52], m6A-SAC-seq [76, 77], MAZTER-seq [53], DART-seq [78], scDART-seq [79], and m6A-REF-seq [59, 80]), direct RNA sequencing [58], and chemical-assisted (GLORI [54]) methods. In total, we retrieved 1928 sequencing runs (FASTQ/FAST5 files) corresponding to 1393 biological samples from GEO [81, 82], GSA [83, 84], and EMBL-EBI [85], covering both *Homo sapiens* and *Mus musculus*. Adapter sequences and low-quality reads were removed using Cutadapt (v.2.9) [86], Trim Galore (v.0.6.6) (https://github.com/FelixKrueger/TrimGalore), and Flexbar 3.0 [87]. PCR duplicates were eliminated via Samtools (v.1.10) [88], FASTX-Toolkit (v.0.0.13) (https://github.com/agordon/fastx_toolkit), and UMI-tools (v.1.1.4) [89]. Fastp (v.0.23.4) [90] performed both trimming and deduplication. For NGS sequencing data, read alignment was performed with HISAT2 (v.2.1.0) [91], HISAT-3N (v.2.2.1–3n-0.0.3) [92], or STAR (v.2.7.11a) [93]. Direct RNA sequencing data were base-called using Guppy (v.3.1.5) and aligned via Minimap2 (v.2.17) [94]. Nanopolish (v.0.13.3) [95] was used to index and align signal events, and m6Anet (v.2.1.0) [58] was used to infer m^6^A sites. Reference genomes were consistently obtained from Ensembl, using GRCh38 (hg38) for human and GRCm38 (mm10) for mouse.

### Standardization of multi-omics data

**Figure 1.**
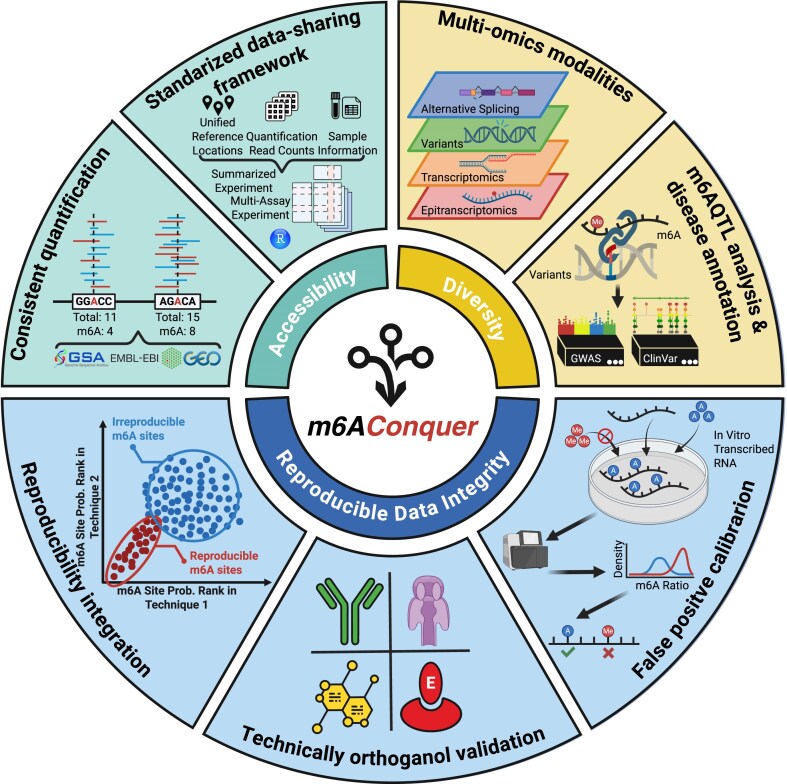
Overall design of m6AConquer.

m6AConquer consistently quantifies data from diverse profiling techniques against reference feature sets to construct standardized matrices (Fig. 1). The database incorporates four primary data types: m^6^A methylomes, expression levels, alternative splicing events, and variant calling results.

### Definition of reference loci

To obtain the methylation quantification matrices, we first defined a broad reference feature set for human (2 788 705 A sites) and mouse (2 906 531 A sites). These sets were constructed by taking the union of all canonical exonic DRACH motifs from the Ensembl v.110 annotation and the non-DRACH/intronic m^6^A sites previously identified by GLORI in the supplementary data of Liu *et al*’s study(54).

### Quantification of m^6^A methylation and transcript expression

All sequencing samples were quantified against the single-based reference feature sets from read alignments without any post-alignment filtering. Methylation signals were consistently summarized using a binomial approach with two measures: (i) m^6^A count: the number of reads capturing methylated adenosines at each locus (m^6^A-specific reads), and (ii) total coverage: the total number of aligned reads from the same sample (or paired input control) representing transcript coverage at that locus. For example, for antibody-assisted techniques (e.g. MeRIP-seq, m6ACE-seq), m^6^A counts were obtained by counting IP read fragments overlapped with the single-based reference A sites using the summarizeOverlaps function from the GenomicAlignments package (v1.40.0). The total coverage was obtained by summing up the counts derived from the IP sample and its paired input sample (counted in the same way as IP). Further details on methylation quantification methods for each sequencing technique are available in the Supplementary Protocol. In addition to the RNA methylome, gene expression levels were quantified from read alignment files of the methylation assays (input samples only for antibody-assisted methods), using Ensembl v.110 gene annotations. RPKM values were computed from raw read counts to normalize the expression levels.

### Alternative splicing detection and variant calling

The alternative splicing matrices employed non-overlapping exonic regions from Ensembl v.110 annotation as reference features. rMATS (v.4.3.0) [96] was used to compute the percent-spliced-in (PSI) scores for five splicing event types (skipped exons, alternative 5′ splice sites, alternative 3′ splice sites, retained introns, and mutually exclusive exons; parameters: –variable-read-length –statoff –individual-counts). SNP data were generated using GATK (v.4.6.1.0) [97] according to the pipeline of the previous study [98]. Variants were called via HaplotypeCaller (-stand-call-conf 20 –minimum-mapping-quality 20) and filtered with VariantFiltration (–filter-name FS -filter “FS > 30.0” –filter-name QD -filter “QD < 2.0”). Only dbSNP-annotated [99] variants, excluding UCSC RepeatMasker microsatellites [100] and REDIportal database [101] entries, were retained.

### Building standardized data-sharing frameworks

The data matrices for all omics modalities were organized as SummarizedExperiment (SE) or RaggedExperiment (RE) objects [102] (RE) with genomic coordinates of reference features (stored in rowRanges) and sample information (stored in colData). For m^6^A SEs, the matrices (accessed through assays()) include m6A counts and total coverage counts for each reference A site (rows) across all samples (columns). These count values have no missing data, making them reliable for benchmarking alternative models or tool fits. Multi-omics integration was performed using MultiAssayExperiment (MAE) [103] to unify the data objects from each profiling technique, ensuring that sample identifiers are consistent across all datasets. Details of the data-sharing frameworks are provided in the Supplementary Protocol.

### IVT calibration and uniform site-calling

After constructing the uniform data-sharing framework, IVT samples were used to calibrate read count matrices for MeRIP-seq, GLORI, and eTAM-seq by masking false-positive sites. IVT samples serve as modification-free negative controls, with gene expression levels matching those of regular mRNA samples [59]. For MeRIP-seq, false positives were identified using the machine learning-based tool m6ACali (v1.0.0) (60), with a false-positive probability threshold set at > 0.5. For GLORI and eTAM-seq, false-positive sites were determined by quantifying IVT samples against the reference m^6^A lites and using a site-calling binomial test with a BH-adjusted *P*-value threshold of < 0.05 (detailed below). To ensure consistency across the dataset, m^6^A-specific read counts and total read coverage at these false-positive sites were set to zero within the data-sharing framework.

After IVT calibration of the read count matrices, we selected the binomial test with Benjamini–Hochberg (BH) correction as the baseline site-calling method for all sequencing techniques. Specifically, we tested for m^6^A enrichment at each site using a one-sided binomial test, comparing the observed m^6^A counts to the expected proportion defined by the average m^6^A modification level across the entire sample (i.e. sum(m^6^A) / sum(Total)). This approach is consistent with the strategies employed by GLORI [54] and eTAM-seq [52]. In parallel, we applied the beta-binomial mixture (BBmix) model as a complementary, probabilistic method to classify m^6^A sites. Originally developed for peak-calling in MeRIP-seq data by MeTPeak [104] and subsequently adopted by MeT-DB2 database [67], BBmix estimates the posterior probability of each site belonging to the m^6^A-enriched foreground. Compared to relying solely on *P*-values, the posterior probabilities generated by BBmix facilitate a clearer distinction between positive (high m^6^A levels) and negative (low m^6^A levels) modification sites at a classification threshold of prob m^6^A > 0.5. Both the BH-adjusted binomial *P*-values and BBmix posterior probabilities are stored as additional assay matrices in the data-sharing frameworks. To ensure data completeness, no fixed *P*-value or posterior thresholds are applied to the quantification matrices; instead, users must define their own thresholds during data analysis. All scripts for the binomial tests and BBmix modeling are available in the reproducible R package OmixM6A, freely accessible at https://github.com/ZW-xjtlu/OmixM6A.

### Reproducibility-based technically orthogonal validation

m6AConquer employs a reproducibility-based approach to integrate quantitative m^6^A profiles across orthogonal sequencing methods. Reproducibility is assessed using the Irreproducible Discovery Rate (IDR) statistical framework in the R package idr (v.1.3) [105], which is the standard method for measuring reproducibility in large-scale ENCODE projects [72]. IDR uses a two-dimensional Gaussian copula mixture model to model the agreement between ranks of paired measurements, assigning each site an IDR score that quantifies the probability it belongs to a reproducible signal component rather than noise. The design of IDR is independent of the threshold choice for each marginal sample, making it suitable for integrating NGS experiments across different platforms, as noted in the discussion section of Li *et al.* [105]. In m6AConquer, the input to IDR was the m^6^A modification levels, expressed as beta-values, which are calculated for each reference site within each technique. The beta-value (${\mathrm{\beta }}$) is defined in Equation 1:


(1) \begin{eqnarray*}
\beta = \frac{{\mathrm{m}}^{6}{\mathrm{A}}}{{\mathrm{m}}^{6}{\mathrm{A}} + {\mathrm{A}}}
\end{eqnarray*}

where “${{{\mathrm{m}}}^{\mathrm{6}}}{\mathrm{A}}$” refers to the number of m^6^A-specific read coverage, and “${\mathrm{A}}$” refers to the unmodified coverage in the corresponding site. The choice of beta-values rather than site-calling *P*-values as input for integration was intended to mitigate confounding by gene expression levels, since highly expressed genes tend to yield smaller *P*-values primarily due to increased coverage rather than proportionally higher m^6^A modification.

To identify orthogonally validated m^6^A sites, integration was performed separately for each pair of techniques classified as technically orthogonal. We defined technical orthogonality as pairs of m^6^A profiling methods originating from distinct biochemical principles: antibody-assisted (MeRIP-seq, m6ACE-seq), enzyme-assisted (MAZTER-seq, eTAM-seq, DART-seq, scDART-seq, m6A-SAC-seq, m6A-REF-seq), chemical-assisted (GLORI), or direct RNA sequencing (ONT-DRS). However, direct RNA sequencing methods and antibody-assisted approaches were considered as non-orthogonal pairs because the m^6^A prediction algorithm used in ONT-DRS (m6Anet(58)) was trained on m6ACE-seq data. Within each technique, m^6^A and total read counts were aggregated across all baseline (non-perturbation) samples to represent the underlying modification state while avoiding biases introduced by experimental perturbations (such as writer knockdowns). For condition-specific analyses, counts were additionally aggregated separately by cell line or tissue type.

For each pair of techniques subjected to IDR integration, beta-values were first computed from the aggregated datasets. To resolve tied beta-values, a jitter factor of 0.0001 was applied using the *jitter* function in R. IDR models were then fitted to beta-values on sites with pairwise high coverage (defined as aggregated total coverage ≥20 in both techniques) to ensure the stability of beta-value estimation in the joint 2D space. The initial parameters for fitting the Gaussian copula mixture model (required in the *est.IDR* function) are set according to the recommended defaults in ENCODE, with mu = 0.1, sigma = 1, rho = 0.2, and *p* = 0.5. To confirm the positivity of the IDR-identified consistent sites, binomial site-calling was also performed on the same aggregated datasets. In the end, a site was defined as orthogonally validated if it satisfied all three of the following criteria: (1) an IDR score < 0.05, indicating high signal agreement between the two techniques; (2) a BH-adjusted *P*-value < 0.05 from the binomial site-calling test in technique 1; and (3) a BH-adjusted *P*-value < 0.05 from the same test in technique 2.

### m^6^A QTL quantification and normalization

To assess the potential roles of m^6^A variation in disease pathogenesis, we performed m^6^A QTL analysis on 211 samples of the human MeRIP-seq dataset following the protocols described by Xiong *et al.* [42, 106]. All selected MeRIP-seq samples in the analysis were from wild-type conditions and used paired-end libraries. Importantly, to ensure the accuracy of findings, we only selected the human orthogonally validated m^6^A sites as the reference sites for m^6^A QTL discovery. For each of the human orthogonally validated sites, sequencing depth-normalized m^6^A modification levels were quantified as log-odds ratios (M-levels), computed as log[(IP / sum(IP)) / (Input / sum(Input))]. Next, sites (matrix rows) with >20% missing values (defined by zero IP + Input total coverage) across samples were removed first. Then, samples (matrix columns) with > 80% missing values across sites were removed. Subsequently, to correct for IP efficiency variability, MA-like normalization was applied as described by Zhang *et al.* [106]. Specifically, for each sample, m^6^A level profiles were compared to a pseudo reference profile (the row-wise mean across all samples), and the difference (M) and average (A) for each site were computed. A linear regression of M against A was fitted, and predicted values were subtracted from the sample profile to remove intensity-dependent biases. Sample-specific GC content biases were further corrected by fitting smoothed linear regressions with natural cubic splines across sites, following the procedure implemented in exomePeak2 [107, 108]. The corrected m^6^A levels were first normalized within each locus (row-wise) by z-score transformation and then quantile-normalized within each sample (column-wise).

### m^6^A QTL detection

Latent batch effects were identified via singular value decomposition (SVD), with the number of factors estimated using the num.sv function from the SVA package [109, 110]. In total, 47 latent factors, together with known batch and cell type annotations, were included as covariates in the QTL analysis. Cis-m^6^A QTL discovery was performed using FastQTL v2.184 [111]. Candidate *cis-*SNPs were restricted to gene bodies (exons and introns defined by Ensembl v110 and promoter regions −2000 bp upstream to + 200 bp downstream of the transcription start site). Empirical *P*-values were derived from 1000 permutations per site, with beta approximation enabled (–permute 1000 –beta). Two stringency thresholds were applied to identify target m^6^A sites: empirical *p* < 0.1 (low) and < 0.05 (high). For each m^6^A site, nominal *P*-value thresholds used to decide the positive QTL lists corresponding to the two thresholds were obtained directly from the FastQTL permutation output (thresholds.txt).

### Disease annotation on m^6^A QTL variants

For disease relevance, variants were annotated with GWAS [112] and ClinVar [113] data following the protocols of the previous study [114]. PLINK v.1.9.0 [115] was used to conduct the linkage disequilibrium (LD) analysis (parameters: –ld-snp-list –ld-window-kb 1000 –ld-window 1 000 000 –ld-window-r2 0.8) with genotype data from the 1000 Genome Project [116–118]. The m^6^A QTL were mapped to these GWAS-associated diseases or traits and ClinVar curated phenotypes separately.

### Orthogonal validation of m^6^A dynamics

orthogonally validated differentially methylated m^6^A sites were identified using the same IDR procedure between orthogonal techniques as described above. For each technique, biological replicates within the same cell type and perturbation condition were aggregated by summing m^6^A and total read counts per site. Only samples under matched perturbation conditions between orthogonal techniques, and sites with total coverage ≥20 in both techniques, were retained for integration. The differences in beta values between treated and control conditions, i.e. (m^6^A_treated / total_treated) − (m^6^A_control / total_control), were used as input for IDR analysis with the idr package (v.1.3) under the same parameter settings in the m^6^A site analysis. Sites with IDR < 0.05 were reported as orthogonally validated differentially methylated sites. At present, such reproducible differential m^6^A methylation sites were successfully identified only in GLORI and eTAM-seq datasets.

### In-depth genomic feature annotation and analysis

Reference sites in m6AConquer were annotated in detail with transcriptomic features, RNA-binding protein (RBP) binding sites, and histone modification profiles to facilitate biological exploration and analysis. Transcriptomic features were defined as combinations of genomic regions (exons, 5′ UTRs, CDS, 3′ UTRs, promoters) and associated properties (length, relative position within the region, distance to 5′ and 3′ ends, and evolutionary conservation level), computed using the genomeDerivedFeatures function from the predictiveFeatures R package [60]. Histone modification data were obtained from ENCODE [119] for germline cell types (H1 for human, ES-E14 for mouse). m^6^A-related RBP data were derived from the m6A_ClipSeqResult datasets in the RM2Target database [120].

To analyze latent patterns among these genomic annotations, we applied a feature embedding and clustering approach conceptually similar to iMPV [121]. Dimensionality reduction was performed on all genomic annotations of the orthogonally validated m^6^A sites, first with principal component analysis (PCA) and then with uniform manifold approximation and projection (UMAP). Hierarchical Density-Based Spatial Clustering of Applications with Noise (HDBSCAN) was then applied to the reduced-dimensionality data, identifying 29 distinct clusters. The cluster annotation was created using an approach similar to scRNA-seq by identifying marker genomic features (analogous to marker genes in scRNA-seq cell clusters) for each cluster. These markers are defined as the features most important for classifying sites within or outside of a cluster, based on SHAP value analysis. For each cluster, a random forest classifier was trained to predict cluster membership of sites (in the cluster or not) using all genomic annotations as the feature input. Annotations were then assigned by interpreting the RF SHAP value diagrams for each cluster and identifying the driving features underlying each module.

### Database and web interface implementation

The m6AConquer platform is built on a MySQL relational database for efficient storage and querying of base-resolution m^6^A methylation data. The web interface was developed using PHP and JavaScript, with HTML, CSS, Bootstrap, and jQuery ensuring a responsive user experience. Interactive data exploration is supported by Data Tables for tabular views and ECharts/HighCharts for dynamic visualizations. Inspired by the GTEx portal, m6AConquer provides multi-dimensional visualization of m^6^A quantification across tissues, cell types, and detection platforms [122]. A customized JBrowse instance enables intuitive navigation of genomic loci with multi-track comparison across techniques [123].

### Quality control reports

We designed a multi-faceted set of quality control (QC) reports to examine sample-level data quality and its site-calling accuracy. Each report begins with a summary table of basic sample characteristics. Next, the GC content bias was evaluated by plotting sites’ GC contents with m^6^A levels. Signal strength was then assessed by clustering quality in the marginal density distributions of m^6^A level. Subsequently, per-site coverage was visualized as the total and m^6^A-modified read depths. PhastCons scores were then used to compare the evolutionary conservation of methylated m^6^A sites versus unmethylated adenosine sites within ±25 bp windows. Expression associations were evaluated by correlating M-levels with gene expression (RPKM). Genomic feature associations were analyzed using logistic regression for site classification and linear regression for methylation level prediction, incorporating known m^6^A-related features (e.g. distance to the nearest exon junction, exon length). Motif associations were quantified using ANOVA-derived R² values computed across 5-mers within DRACH motifs. Finally, the statistical assumptions behind different site-calling methods were benchmarked by assessing prediction accuracy on OV sites via Matthews Correlation Coefficient (MCC) and empirical fits using quantile–quantile plots. QC reports for all samples are available on the download page of the m6AConquer website.

## Results

### An integrated and orthogonally validated m^6^A methylome

**Figure 2.**
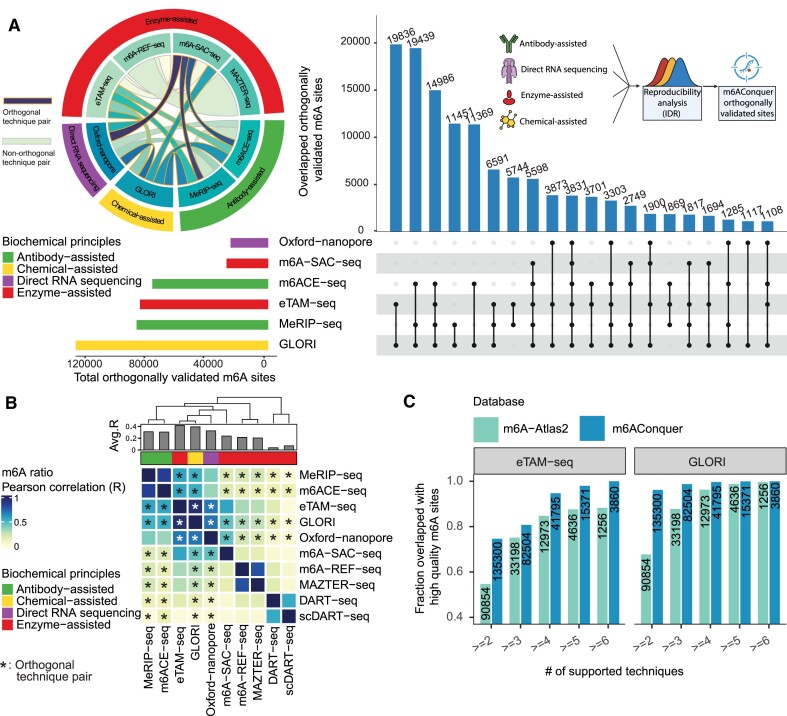
Overview of the consistently quantified and orthogonal validated methylome​ in m6AConquer. (**A**) Landscape of orthogonally validated m^6^A sites. The UpSet plot (top right) visualizes the intersections of reproducible m^6^A sites (IDR < 0.05) validated across techniques with distinct biochemical principles. The horizontal bar plot (bottom left) shows the total number of unique validated sites identified with each key technology. The chord diagram (top left) illustrates the number of reproducible sites identified between any pair of techniques, with links between technically orthogonal pairs highlighted. A schematic (inset) depicts the overall validation workflow. (**B**) Pairwise Pearson correlation of m^6^A ratios (beta-values) across 10 technologies. Ratios were calculated from aggregated data within the technique. Technique categories are color-coded, and asterisks (*) denote technically orthogonal pairs. (**C**) The fraction of multi-technique supported sites verified by high-quality m^6^A sites (defined as sites also identified by eTAM-seq or GLORI). The comparison is made between sites from m6AConquer (identified by orthogonal validation) and m6A-Atlas2 (identified by simple intersection). The numbers on the bars indicate the absolute count of sites at each support level, demonstrating the superior reliability of the m6AConquer set.

m6AConquer is an integrated data platform that compiles quantitative m^6^A sequencing data from 1393 human and mouse samples, obtained through ten different profiling techniques. To facilitate consistent cross-dataset analyses, we defined approximately 2.8 million reference adenine sites each for human ([2],788 705) and mouse ([2],761,[121]) genomes. Following this, we consistently quantified methylation ratio (as beta-value) across 11 distinct m^6^A detection techniques over the same reference sites. Our initial comparative analyses indicated moderate agreement in methylation profiles among most m^6^A detection methods, with GLORI and eTAM-seq exhibiting the highest correlation (Pearson’s *r* = 0.96, Fig. 2B).

To reliably integrate data from multiple m^6^A profiling techniques, we applied the IDR framework to quantify reproducibility among orthogonal techniques, each based on distinct biochemical principles. This approach follows the methodology used in the ENCODE project for large-scale epigenetic data integration. [72]. Using this reproducibility-focused strategy, we identified 135 300 orthogonally validated m^6^A sites (IDR < 0.05) in human samples, of which 61% (82 504 sites) were further supported by at least two distinct pairs of sequencing methods. GLORI and eTAM-seq showed the highest reproducible sites (71 736 sites) among all orthogonal technique pairs (Fig. 2A). Compared to m6A-Atlas V2.0, which uses a simpler intersection-based strategy typical of established databases, our reproducibility- and orthogonality-driven approach identified a greater fraction (and number) of GLORI and eTAM Seq-confirmed sites across all thresholds (Fig. 2C). This enhanced accuracy highlights the value of our de novo quantification and statistical integration strategy, which helps mitigate method-specific artifacts that could confound simpler intersection-based approaches and those that overlook technical dependencies. Additionally, validation using these orthogonally validated sites as ground truth in benchmarks demonstrated strong performance of eTAM-seq (AUC = 0.99) and GLORI (AUC = 0.98) for site prediction (Supplementary Fig. S1). We further stratified this benchmark by DRACH motifs and other sequence contexts, and showed that our reference sites are not particularly biased toward GLORI (Supplementary Fig. S4). These results demonstrate the practical value of reproducibility-based data integration across independent m^6^A profiling techniques with different bio-chemical principles.

### The m6AConquer web portal and usage

#### Querying and visualizing m^6^A sites for a gene of interest

**Figure 3.**
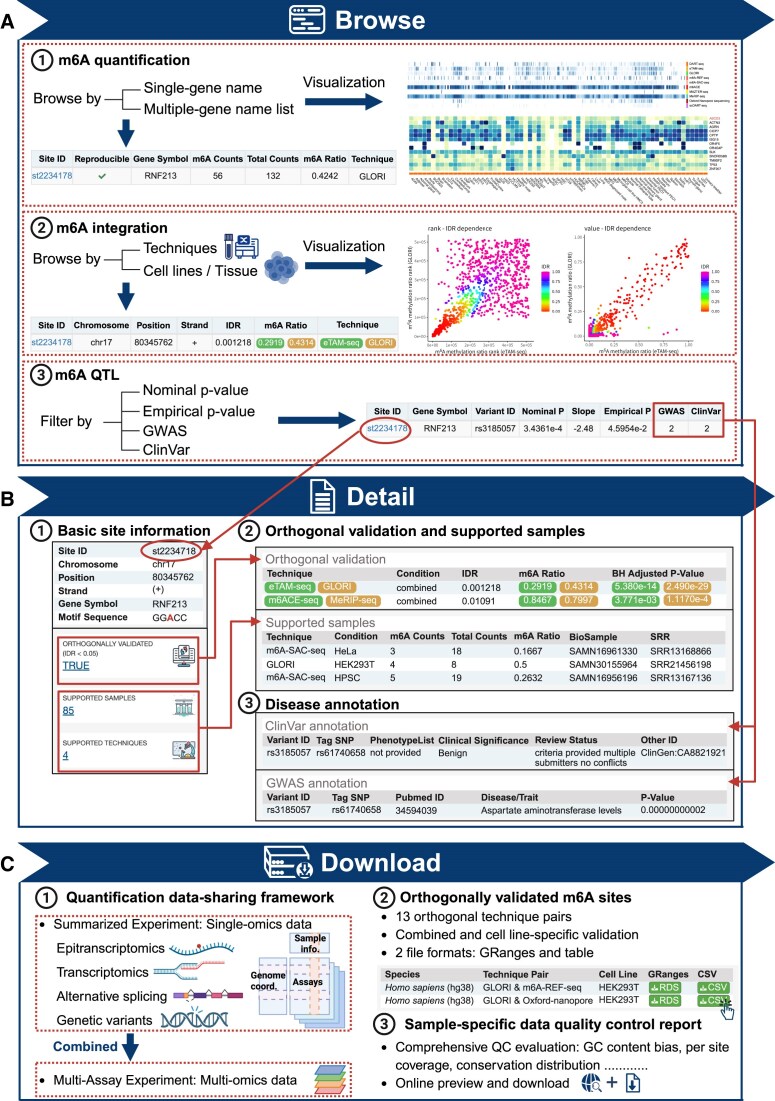
Interactive query interface of the m6AConquer website. (**A**) m6AConquer provides data browsing for m^6^A quantification, m^6^A integration, and m^6^A QTL data with various filters and data visualization. (**B**) Clickable Site IDs (e.g. st2234178) access detailed information, including Motif sequences (e.g. “TGACT”), Supporting techniques/samples, Orthogonal reproducibility status, and Disease annotation. (**C**) Download module in m6AConquer contains standardized data-sharing framework (single-omics and multi-omics), orthogonally validated m^6^A sites, and a sample-specific data quality control report.

m6AConquer provides gene-based queries of collected m^6^A quantitative profiles (Fig. 3A). For example, users can query and browse for m^6^A coordinates and abundances located at gene *RNF213*, which is widely recognized as the major genetic susceptibility factor for Moyamoya disease (MMD), using the gene symbol name in the search box. Detailed information of each queried m^6^A site can be accessed through the link on site ID (e.g. st2234178), such as motif sequences, supported sequencing samples and techniques, and orthogonal integration result (Fig. 3B). The m6AConquer also supports the multi-gene query by entering a list of gene symbol names. Various filters, including profiling techniques and sample cell lines or tissues, allow users to collectively explore m^6^A quantitative profiles of interest. Real-time generated m^6^A level distribution plots and heatmaps are presented as data visualization to analyze the clustering pattern of m^6^A levels among candidate genes (Fig. 3A).

### Exploring reproducibility across different technologies

**Figure 4.**
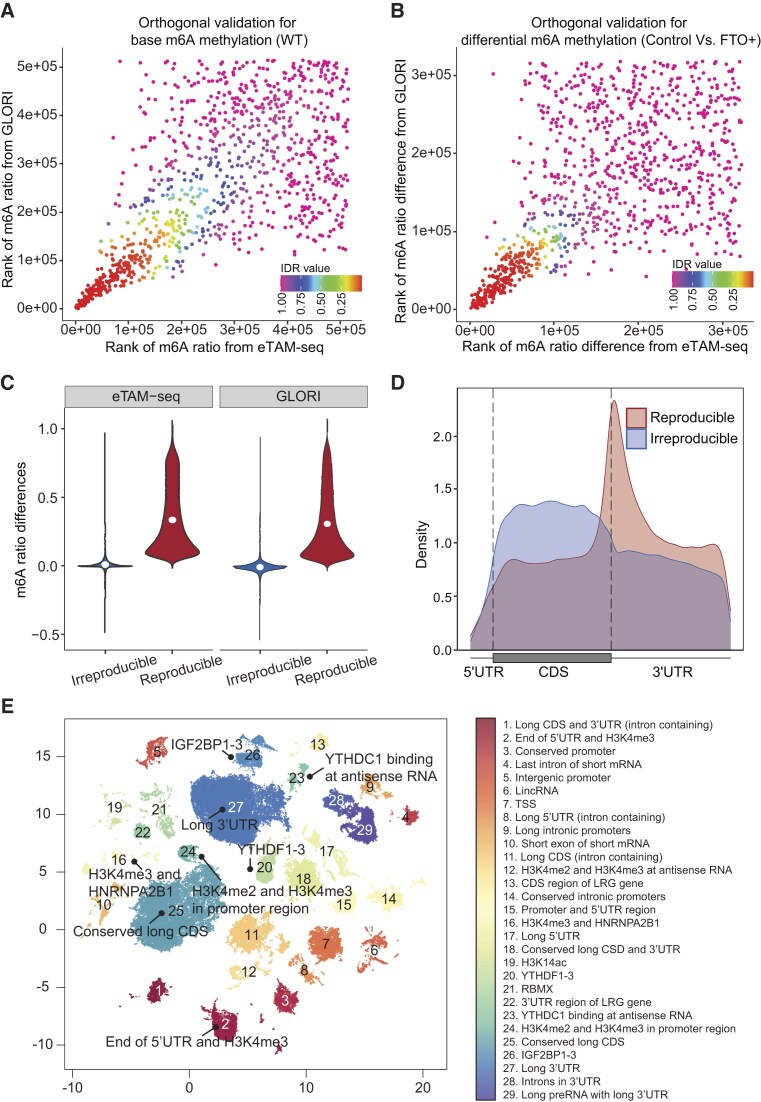
Orthogonal validation reveals robust and functionally stratified m^6^A sites. (**A**) Rank-rank scatter plot of m^6^A ratio (beta-value) between eTAM-seq and GLORI, colored by IDR. Axes represent the site ranks from each technique. (**B**) Rank-rank scatter plot of m^6^A ratio differences between control and *FTO*-treated in-vitro RNA samples. Axes represent the ranks of differences from each technique. Scatter plots in A and B are downsampled to 1000 points for visualization clarity. (**C**) Comparing m^6^A ratio differences for reproducible vs. irreproducible differentially methylated sites as determined by IDR < 0.05 in Fig. 4B. Reproducible sites show larger differences. (**D**) Transcript topology profile of the orthogonally validated *FTO* responsive sites (*n* = 62 002), demonstrating enrichment near stop codons. (**E**) Unsupervised clustering of orthogonally validated m^6^A sites in human based on their genomic features. A two-dimensional UMAP projection was generated from 184 genomic annotations associated with each orthogonally validated m^6^A site. HDBSCAN was then applied to the UMAP embedding to identify distinct clusters. The plot displays 29 identified clusters, with cluster labels indicating the driving genomic annotations for each cluster. The cluster label was obtained from SHAP value analysis of random forest classifiers, which predicted whether a site belongs to a given cluster using 184 genomic annotations. As a result, the driving factors identified for each cluster include transcript features (e.g. long 3′UTRs), promoter-associated chromatin marks (e.g. H3K4me3), and RNA-binding protein (RBP) interaction hubs (e.g. *IGF2BP1-3*). This stratification reveals the diverse regulatory landscapes where m^6^A modification occurs and suggests that distinct functional roles are governed by the local genomic context.

The m^6^A integration module contains the orthogonal validation results between pairs of techniques (Fig. 3A). Users can browse for validated m^6^A sites by the technique pairs and biological conditions (optional) of interest, retrieving the corresponding reproducible m^6^A site visualization plots and result tables. The result tables can be directly downloaded as a CSV file. The specific case of IDR integration outcomes between GLORI and eTAM-seq is presented in this manuscript (Fig. 4A). A bootstrap down-sampling simulation further indicates that model fitting and outcomes remain stable for these two orthogonal techniques, even at low sequencing depths (Supplementary Fig. S3).

### Linking m^6^A variation to human disease

The m^6^A QTL module contains the m^6^A QTL mapping results and the corresponding disease annotations. Under the high stringency criteria (empirical *p* < 0.05), 6034 m^6^A QTL were identified in m6AConquer, with 428 m^6^A QTL annotated by ClinVar and 1763 m^6^A QTL annotated by GWAS. Users can filter and browse the m^6^A QTL results with target m^6^A locations, variant RS IDs, slopes and *P*-values (Fig. 3A). Detailed variant information and disease annotation can be accessed through the link on the site ID (Fig. 3B). To further assess the quality of our m6A QTL dataset, we selected m6A QTLs whose associated variants lie within the DRACH motif of the corresponding m6A sites. We observed that variants disrupting the DRACH motif have significantly more negative m6A QTL effect sizes (slopes) compared to those that do not (t-test *p* = 0.00775). It is worth noting that, in our dataset, all observed slopes for DRACH-disrupting variants were negative (Supplementary Fig. S2).

### Convenient and multi-modal data download

m6AConquer provides multi-modal data for download (Fig. 3C). Firstly, our web server allows the export of both single-omics and multi-omics data-sharing frameworks for omics quantification data. Secondly, the orthogonally validated m^6^A sites are also available in both R Genomic Ranges files (in RDS format) or tabular text files (in CSV and BED format). Both combined and cell-line-specific integration are provided. Lastly, users can also retrieve and download the quality control reports for each sample. To facilitate ease of use, the m6AConquer web portal is accompanied by extensive documentation, step-by-step tutorials, and detailed guides on its data-sharing frameworks.

### Case Study 1: Identifying orthogonally validated differential methylation

To demonstrate its utility in studying m^6^A dynamics, m6AConquer supports the identification of orthogonally validated, differentially methylated sites. As a case study, we investigated changes in m6A methylation following the in-vitro demethylase treatment of RNA with *FTO*, using data from the technically independent GLORI and eTAM-seq methods. We first calculated the change in methylation levels (beta-value differences) for sites with sufficient coverage (≥20 reads). Applying our IDR statistical framework to these differential methylation effect sizes (Fig. 4B), we successfully identified 62 002 reproducible sites of differential methylation (IDR < 0.05). Interestingly, these orthogonally validated *FTO*-responsive sites showed a distinct enrichment near the stop codon (Fig. 4D), consistent with established basal m^6^A topology. The validated differential sites also exhibited substantially larger changes in methylation ratio compared to irreproducible sites (Fig. 4C).

### Case Study 2: Uncovering regulatory contexts of m^6^A sites via unsupervised clustering

To showcase the potential of the rich set of genomic annotations curated in m6AConquer, we performed an unsupervised clustering of genome features on the orthogonally validated m^6^A sites in human. All necessary data for this analysis, including site coordinates and 184 genomic features spanning transcript topology, RBP binding, and histone modifications, were exported directly from m6AConquer’s annotation dataset. Dimensionality reduction via UMAP revealed clear patterns of functional organization, stratifying the m^6^A sites into 29 distinct clusters based on their genomic context (Fig. 4E).

Interestingly, we further found that each cluster was driven by a distinct combination of features, potentially uncovering their diverse regulatory roles. For instance, Cluster 2, characterized by proximity to the 5′UTR end and co-occurrence with H3K4me3, suggests a role in transcriptional initiation. In contrast, Cluster 27 represents sites within long 3′UTRs, implicating them in mRNA stability. The analysis also identified key “m^6^A hubs” defined by the co-localization of specific reader proteins, such as *YTHDF1-3* (Cluster 20) and *IGF2BP1-3* (Cluster 26). It also identified m^6^A sites bound by histone modifications H3K4me2 and H3K4me3, which are associated with the promoters of genes containing evolutionarily conserved CDS (Cluster 24 and Cluster 25). This case study demonstrates that the multi-layered, detailed genomic annotation data within m6AConquer provide a useful resource for dissecting the complex regulatory landscapes of m^6^A.

## Discussion

The rapid progress in epitranscriptomic sequencing has created both immense opportunities and a critical challenge: the lack of reproducibility and coherent integration of data from disparate sources. In this work, we developed m6AConquer to address this challenge by replacing simple data aggregation with consistent re-quantification and integration strategies grounded in reproducibility. Our approach leverages the rigorous IDR framework to assess concordance across orthogonal techniques, thereby establishing a high-confidence **reference m**^**6**^**A methylome**. This integration strategy effectively filters out technique-specific artifacts and spurious signals, a conclusion supported by its observed improvement in accuracy over a simple intersection-based m^6^A database and by the high performance of absolute quantitative techniques (eTAM-seq and GLORI) evaluated in our curated benchmark dataset.

A central goal of m6AConquer project is to facilitate the exploration of m^6^A’s functional roles. By providing matched multi-omics data within a unified data-sharing framework, the platform supports researchers to connect methylation events to downstream consequences. For example, our m^6^A QTL analysis of m^6^A sites validated by orthogonal methods illustrates how users can reliably connect genetic variation to RNA methylation, generating testable hypotheses about m^6^A dysregulation in human disease.

Beyond functional genomics, our consistent data-sharing framework offers a highly valuable resource to the computational research community. While challenges such as batch effects, technical artifacts, and distributional shifts can confound tool and model evaluations, as seen in the ENCODE imputation challenge [124], m6AConquer directly mitigates these issues through its rigorous, consistent, and analysis-ready data curation. Crucially, our platform provides complete and unbiased quantification matrices that include both methylated and unmethylated sites. Together, these features address the data sparsity and selection bias inherent in “positive-call-only” databases, offering an ideal, high quality substrate for training and benchmarking generalizable AI models.

The m6AConquer project currently faces several limitations. First, it does not fully eliminate heterogeneity arising from the upstream processing steps of different m^6^A sequencing methods, as the initial pipelines before quantification and site-calling (e.g. trimming and read alignment) still follow the standard protocols from each technique’s original publication. Second, although it applies consistent quantification procedures and IVT calibration wherever possible, it cannot fully remove technique-specific artifacts caused by detection limitations, such as the low sequencing depth in ONT-based methods and the highly skewed profiles generated by non-absolute quantification techniques. Future studies should therefore focus on automating data standardization through reproducible software workflows implemented using Docker containers and on developing additional ML tools that leverage IVT control samples to calibrate false positives across diverse sequencing technologies.

In conclusion, m6AConquer offers the scientific community a rigorously curated dataset and a transparent strategy for m^6^A analysis. While the current work prioritizes quality over quantity, future developments aim to achieve both. The dataset will grow in parallel with the field’s emphasis on high-quality, orthogonal data from absolute quantification methods (e.g. GLORI, eTAM-seq) and emerging chemical conversion–based techniques such as m6A-CAM-seq [125]. As these results accumulate, their integration will enable the construction of a far more detailed map of validated m^6^A profiles, allowing for high-resolution analysis of m^6^A dynamics across diverse cell types and perturbations. By providing an consistently quantified and orthogonally validated atlas of the m^6^A methylome, we hope this resource will serve as a practical *ground truth* for computational benchmarking and enhance the reliability of continued advances in RNA methylation research.

## Supplementary Material

gkaf1204_Supplemental_Files

## Data Availability

The m6AConquer database is freely available to all users without any login requirement at https://rnamd.org/m6aconquer/. The source code for processing raw data in m6AConquer is provided at GitHub (https://github.com/XichenZhao0223/m6AConquer-Data-Processing) with an accompanying Zenodo archive (DOI: 10.5281/zenodo.17169694). The source code for the binomial/BBmix site-calling is available in the reproducible R package OmixM6A at GitHub (https://github.com/ZW-xjtlu/OmixM6A) and archived at Zenodo (DOI:10.5281/zenodo.13337113). The curated data matrices in SummarizedExperiment and MultiAssayExperiment formats are available for batch download from the database website.
