# Supplementary material for: m6AConquer: a consistently quantified and orthogonally validated database for the N6-methyladenosine (m6A) epitranscriptome
Source: Nucleic Acids Res. 2025 Dec 3;54(D1):D204–18. doi: 10.1093/nar/gkaf1204 (PMC12807759; doi:10.1093/nar/gkaf1204)
Supplement: gkaf1204_Supplemental_Files [file gkaf1204_supplemental_files.zip › Supplementary Protocol.pdf]

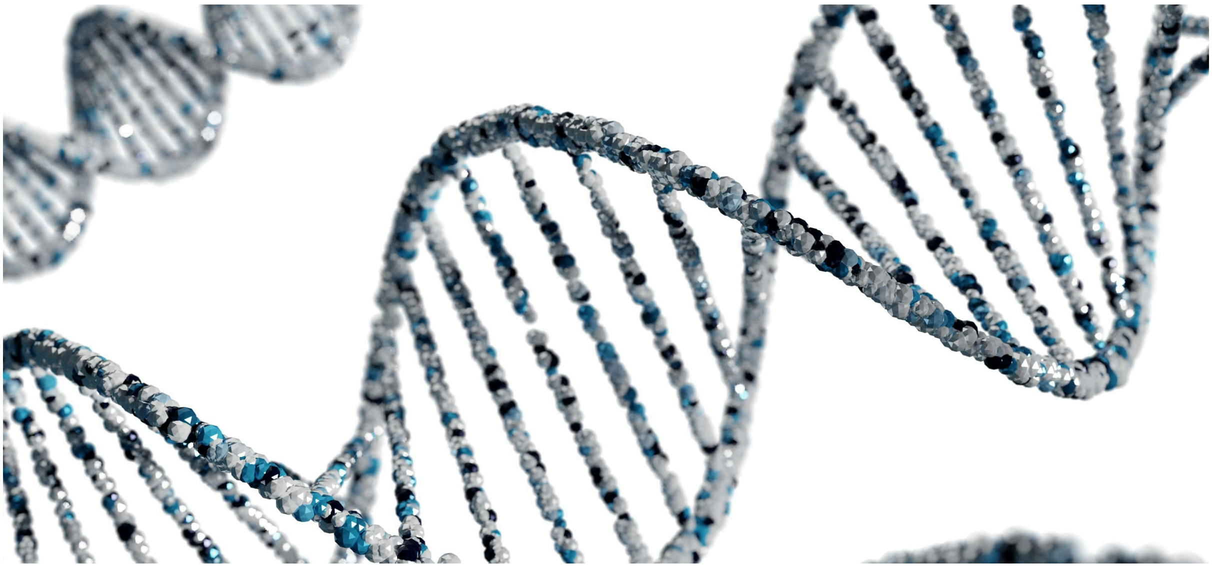

Supplementary Protocol

Content:

Project Introduction
Reference Tables for Sources and Tools
- Reference genome
- Data sources
- Tools in raw data processing
- Tools in downstream analysis

m6AConquer Protocol
- 1. Sequencing data acquisition and quality filtering
- 2. Read alignment and mapping optimization
- 3. Epitranscriptomic data processing and integration
  - **3.1. Feature counting**
  - **3.2. IVT calibration and QC reports**
  - **3.3. Site calling**
  - **3.4. Orthogonal validation of m6A detection techniques**
- 4. Data processing and integration for other omics modalities
  - 4.1. Transcriptomic data processing and analysis
  - 4.2. Alternative splicing quantification and profiling
  - 4.3. Genetic variant analysis and interpretation
- 5. Build of standardized data-sharing frameworks
- 6. m6A QTL analysis and disease association

Project Introduction

The rapid growth of sequencing-based methods to detect RNA N6-methyladenosine (m6A) modifications has introduced substantial reproducibility challenges. Differences in experimental protocols and computational analyses complicate cross-study comparisons, obscuring accurate characterization of the m6A landscape. Existing databases aggregate pre-processed data, retaining biases specific to each profiling technology without a consistent validation framework.

To address these issues, we developed **m6AConquer**, a comprehensive database emphasizing reproducible quantification and integration. Raw data from ten profiling technologies—including GLORI and eTAM-seq—were uniformly reprocessed, quantifying methylation at over **2.7 million reference sites** in human and mouse.

Our standardised pipeline features false-positive calibration using **in-vitro transcribed (IVT) controls** and consistent statistical site-calling criteria. A reproducibility-based integration **leveraging technical orthogonality** among profiling methods identified over **135,000 high-confidence m6A sites (IDR < 0.05)**.

Additionally, m6AConquer provides matched multi-omics data (expression, splicing, genetic variants) and **cis-m6A quantitative trait loci (m6A-QTLs)**, facilitating investigation into genetic regulation and disease associations. The database includes intuitive query interfaces, interactive visualisations, and **analysis-ready data objects**, supporting reproducible and transparent m6A research.

Reference Tables for Sources and Tools

Reference genome

Organism	Reference genome	URL
Homo sapiens	hg38/GRCh38	https://hgdownload.soe.ucsc.edu/downloads.html#human; https://www.ensembl.org/Homo_sapiens/Info/Index
Mus musculus	mm10/GRCm38	https://hgdownload.soe.ucsc.edu/downloads.html#mouse; https://www.ensembl.org/Mus_musculus/Info/Index

Data sources

Technique	Source	URL
GLORI	GEO	https://www.ncbi.nlm.nih.gov/geo/
DART-seq	GEO	https://www.ncbi.nlm.nih.gov/geo/
eTAM-seq	GEO	https://www.ncbi.nlm.nih.gov/geo/
m6A-REF-seq	GEO	https://www.ncbi.nlm.nih.gov/geo/

Technique	Source	URL
m6A-SAC-seq	GEO	https://www.ncbi.nlm.nih.gov/geo/
MAZTER-seq	GEO	https://www.ncbi.nlm.nih.gov/geo/
scDART-seq	GEO	https://www.ncbi.nlm.nih.gov/geo/
MeRIP-seq	GEO; GSA	https://www.ncbi.nlm.nih.gov/geo/; https://ngdc.cncb.ac.cn/gsa/
m6ACE-seq	GEO	https://www.ncbi.nlm.nih.gov/geo/
Oxford-nanopore	EBI	https://www.ebi.ac.uk/

Tools in raw data processing

Techniques	Quality Control	Alignment
GLORI	**Trim Galore v.0.6.6, Fastp v.0.23.4**	**STAR v.2.7.11a, bowtie v.1.3.1**
DART-seq	**Fastp v.0.23.4**	**HISAT-3N v.2.2.1-3n-0.0.3 (C → T)**
eTAM-seq	**Cutadapt v.2.9, UMI-tools v.1.1.4**	**HISAT-3N v.2.2.1-3n-0.0.3 (A → G)**
m6A-REF-seq	**Cutadapt v.2.9**	**HISAT2 v.2.1.0**
m6A-SAC-seq	**Cutadapt v.2.9, Fastx_collapser v.0.0.13**	**STAR v.2.7.11a**
MAZTER-seq	**Trim Galore v.0.6.6**	**STAR v.2.7.11a**
scDART-seq	**Flexbar**	**STAR v.2.7.11a**
MeRIP-seq	**Trim Galore v.0.6.6**	**HISAT2 v.2.1.0**
m6ACE-seq	**Fastp v.0.23.4**	**STAR v.2.7.11a**
Oxford-nanopore	**Guppy v.3.1.5 (Basecalling)**	**Minimap2 v.2.17**

Tools in downstream analysis

Tool	Description	URL
m6ACali	MeRIP-seq data false positive calibration	https://github.com/HaokaiYe/m6ACalibrateR_manuscript
rMATS (v.4.3.0)	Percent-spliced-in (PSI) score calculation	https://github.com/Xinglab/rmats-turbo
GATK (v.4.6.1.0)	Variant calling and filtration	https://github.com/broadinstitute/gatk
FastQTL (v.2.184)	Cis-m6A QTL mapping	https://github.com/francois-a/fastqtl
PLINK (v.1.9.0)	Linkage disequilibrium calculation	https://www.cog-genomics.org/plink/

Tool	Description	URL
m6Anet (v.2.1.0)	m6A detection from DRS	https://github.com/GoekeLab/m6anet

m6AConquer Protocol

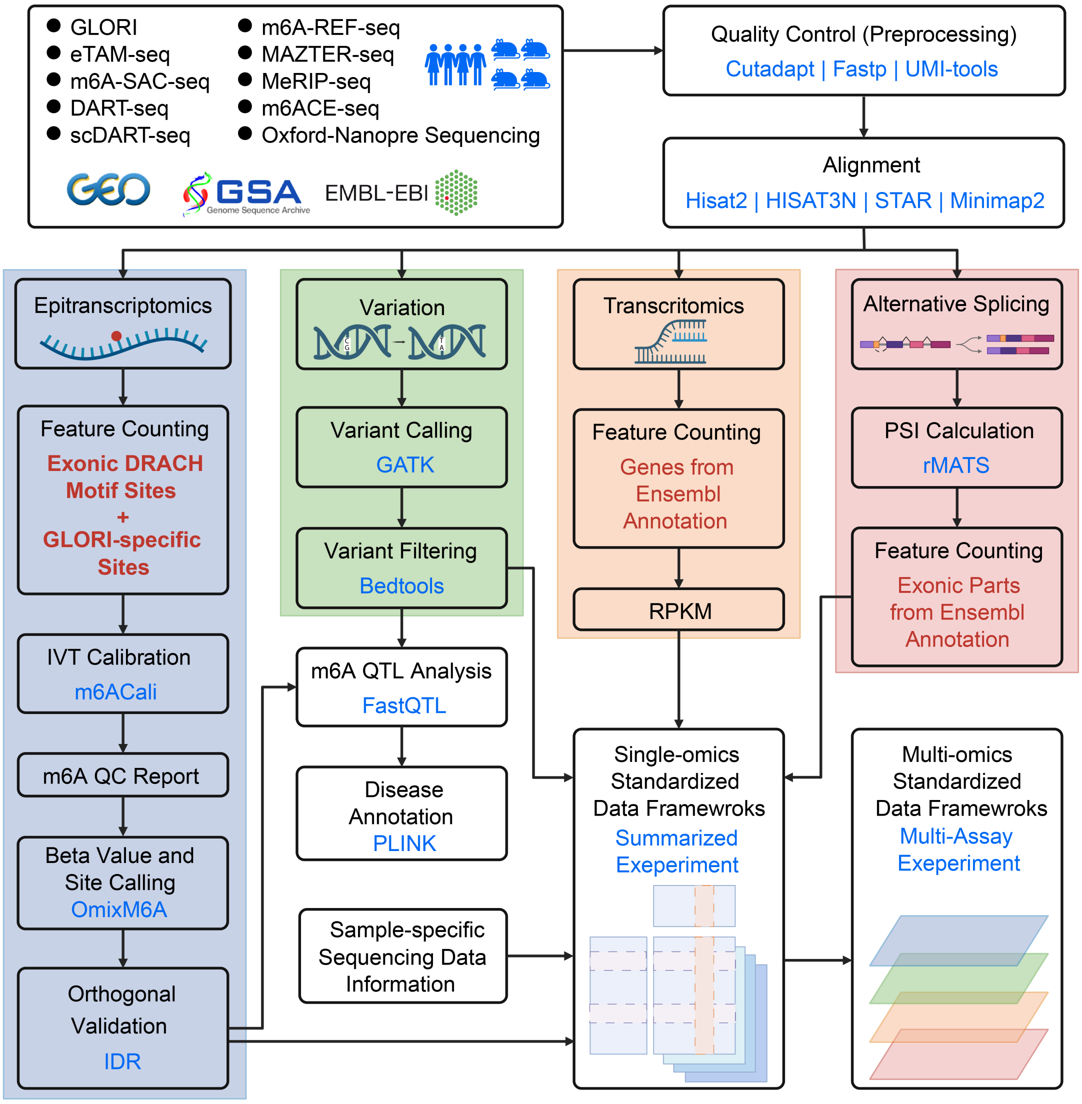

Workflow of m6AConquer

1. Sequencing data acquisition and quality filtering

m6AConquer collects external raw sequencing data from **10 m6A profiling techniques,** including GLORI, eTAM-Seq, m6A-SAC-Seq, DART-Seq, scDART-Seq, m6A-REF-Seq, MAZTER-Seq, MeRIP-Seq, m6ACE-Seq, and ONT DRS. For MeRIP-Seq data, we select only paired-end library types to ensure the consistency of the downstream analysis. Additionally, all available IVT negative control samples are collected to enable calibration for false-positive m6A loci. Samples from both wild-type and perturbation

conditions (e.g. in-vivo m6A regulators knockdown or heat shock stress treatment) are included in the database.

FASTQ files for NGS techniques are retrieved and downloaded accordingly from GEO, GSA, and EMBL-EBI. NGS samples underwent initial read trimming using tools including **Cutadapt**, **Trim Galore**, **Flexbar**, **Fastp** to cut out adaptors and low-quality reads. After trimming, for samples of some techniques, raw reads are deduplicated through **Samtools**, **Fastx Collapser**, **UMI-tools**, and **Fastp** to remove PCR duplications. As for ONT DRS data, the raw Fast5 files are obtained from the **Singapore Nanopore-Expression Project**. Quality control is conducted during the basecalling process with **Guppy**. Please refer to **this Github page** for detailed processing methods for each type of technique.

2. Read alignment and mapping optimization

For NGS sequencing data, 4 aligners (**STAR**, **Bowtie**, **HISAT2**, and **HISAT-3N**) are collectively utilized for different techniques based on their original pipelines. Bowtie is employed explicitly for transcriptome alignment, while the other three aligners are utilized for genome alignment. The genome index of **HISAT2** can be obtained from this link: **https://genome-idx.s3.amazonaws.com/hisat/grch38_genome.tar.gz**.

Meanwhile, the genome indexes for **STAR** and **HISAT-3N** are constructed as per the specific requirements. All sequencing reads are aligned to the GRCh38 or hg38 human genome, and various versions of gene annotation from **RefSeq**, **Genecode**, and **Ensembl** are employed under their original pipelines.

In the context of ONT DRS, the aligner **minimap2** is utilized for transcriptome alignment. The Ensembl transcript reference of GRCh38 is indexed using the default parameters of **minimap2**. The m6A stoichiometry for the ONT DRS datasets after alignment are called using **m6Anet**. The codes for the index building and alignment for each technique can be accessed on our data processing GitHub page.

3. Epitranscriptomic data processing and integration

3.1. Feature counting

To obtain the m6A methylome quantification matrices, we defined the reference features as the A sites from 2,526,220 human and 2,666,051 mouse exonic DRACH motifs, based on Ensembl v110 (human) and v102 (mouse) annotations. In addition, non-DRACH and intronic sites were included by including 262,485 human and 240,480 mouse GLORI-identified m6A sites from **Cong's study** outside of exonic DRACH motifs.

m6A-specific read counts and total RNA coverage were quantified from aligned sequencing data at base-resolution reference sites, without applying any post-alignment filtering. For example, for antibody-dependent techniques (MeRIP-seq and m6ACE-seq), m6A counts were obtained from the # of IP read fragments that overlap

the single-based reference A sites, which is calculated by the `summarizedOverlaps` R function (`fragments = TRUE`) in **GenomicAlignments**. The total coverages were then calculated by combining the read counts from both the IP and the paired input samples. For details about how m6A and total coverage counts were defined for each m6A detection technique, please refer to the reference table in the m6AConquer **Data Framework Usage Tutorial** page.

3.2. IVT calibration and QC reports

m6AConquer collected all **in vitro transcription (IVT) negative control samples** from GLORI, eTAM-seq, and MeRIP-seq datasets. IVT control samples are modification-free while having the same gene expression level as regular mRNA samples.

To calibrate m6A sites using the negative controls, the binomial test (see 3.3 Site calling) is used to call false positive sites from the combined IVT control sequencing samples from GLORI and eTAM-seq . Then, sites with a BH-adjusted binomial test p-value < 0.05 are considered false positive m6A sites for the respective GLORI or eTAM-seq datasets (see 3.3 for site calling details). In the case of samples from MeRIP-seq, machine learning based method of **m6ACali** is used to identify false positive m6A sites at a threshold of FP prob > 0.5. Subsequently, for each mRNA sequencing sample in these three techniques, the m6A counts and total counts at identified false-positive m6A sites were set to zero, thereby masking them in our quantification matrices and excluding them from downstream analyses.

In addition, we implemented a comprehensive quality control (QC) reporting system to evaluate both data quality and site-calling accuracy. These QC reports are generated from the data matrices after IVT-based filtering, and the results for all samples are available on our **Quality Control Report** page.

3.3. Site calling

After IVT calibration, m6AConquer performed site calling across all reference loci to identify significantly methylated m6A sites. For each sample, we applied a one-sided binomial test with the null hypothesis that the site-specific methylation ratio is less than or equal to the global background ratio, defined as the total m6A reads divided by the total coverage across all reference sites in that sample. The p-values were computed in R using the `pbinom` function with `lower.tail = FALSE`, and then adjusted for multiple testing using the Benjamini–Hochberg procedure (`p.adjust`, method = "BH").

In addition, we also used a two-component beta-binomial mixture (BBmix) method as an alternative probabilistic site calling strategy. This method originated from **MetPeak** for MeRIP-seq peak calling and was implemented in **MeT-DB2**. BBmix estimated the posterior probability of each site assigned to the m6A-enriched foreground. Users can select positive m6A sites with a BBmix posterior threshold of ≥ 0.5, while sites with a posterior < 0.5 correspond to negative sites.

Importantly, we did not apply any filtering based on the adjusted p-value or posterior in our matrices within the data-sharing frameworks; instead, we store the adjusted p-value and BBmix posterior in the framework as additional matrices to allow users to decide their own thresholds. For detailed implementation and options for customizing our site-calling strategies, please refer to our reproducible R package ***OmixM6A***.

3.4. Orthogonal validation of m6A detection techniques

To identify reliable modification sites and quantify consistency between m6A detection techniques, m6AConquer applies the **Irreproducible Discovery Rate (IDR)**, a reproducibility-based statistical integration framework for high-throughput omics experiments. IDR has been widely adopted in projects such as ENCODE to identify epigenetic peaks that are consistently observed across replicates. The statistical framework can be easily used to quantify consistency between different NGS platforms, as mentioned in the discussion section of **Qunhua et al's paper**. In our implementation, we used IDR to quantify the consistency between different m6A profiling techniques, particularly between those employing **orthogonal biochemical principles** to measure m6A abundances.

To begin with, we classified the 10 m6A quantitative profiling techniques into **4 orthogonal groups** based on their biochemical principles:

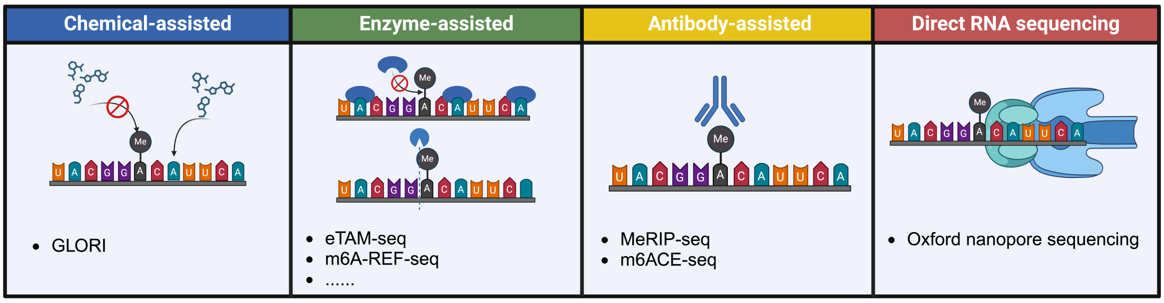

4 orthogonal sequencing strategies classified for m6A quantitative profiling techniques

- **Chemical-assisted**: Converts unmodified adenosines to alternative bases (e.g., inosine) through chemical reaction, while m6A-modified adenosines remain resistant to conversion.
- **Enzyme-assisted:** Utilizes enzymes whose activity depends on m6A presence, such as endonucleases that cleave only unmethylated motifs, enabling m6A site discrimination.
- **Antibody-assisted**: Enriches RNA fragments carrying m6A modifications via antibody-based immunoprecipitation, typically providing regional (rather than single-nucleotide) resolution.

- **Direct RNA sequencing**: Detects m6A modifications directly from native RNA molecules using Oxford Nanopore sequencing, where modified adenosines produce characteristic current signal shifts.

Subsequently, we defined technical orthogonality as comparisons between methods from different categories (antibody-assisted, enzyme-assisted, chemical-assisted, or direct RNA sequencing, DRS). An exception was made for DRS and antibody-assisted methods, which were not considered orthogonal because our DRS site calling relied on ***m6Anet***, a model trained with antibody-based m6ACE-seq data as ground truth.

For each technique, m6A and total coverage counts were aggregated across all baseline samples, excluding perturbation conditions such as writer knockdown/knockout, in order to capture the basal m6A pattern. For each orthogonal pair of techniques, only sites with pooled total coverage ≥20 in both datasets were retained to minimize noise from low expression. Next, we compute the methylation ratio at each site (m6A reads / total coverage, i.e., the beta-value) for each technique as the input for IDR analysis.

IDR analysis was then implemented through the ***idr*** R package. Sites were considered orthogonally validated if they satisfied both IDR < 0.05 and significant enrichment (i.e. site called) by the binomial test (BH-adjusted p < 0.05) in each technique. Applying these criteria, we identified 135,300 validated sites in human. All validated sites are provided for direct **download** in the database.

4. Data processing and integration for other omics modalities

4.1. Transcriptomic data processing and analysis

m6AConquer also quantifies gene expression with the `summarizedOverlaps` R function from all RNA sequencing datasets used in m6A profiling analyses, generating expression level matrices for 58,560 human genes and 55,401 mouse genes based on Ensembl annotations (v110 for human, v102 for mouse; the latest available at the time of processing). For MeRIP-seq datasets, only input samples (excluding IP libraries) were used, as input libraries best represent transcript abundance. Both raw read counts and normalized values (RPKM) were computed and stored within the SummarizedExperiment (SE) objects, accessible through the `assays()` function.

4.2. Alternative splicing quantification and profiling

To profile alternative splicing, we used exon–intron structures defined by Ensembl v110 (human) gene annotation. From this annotation, 671,257 disjoint exonic regions were generated as the reference coordinate set. Alternative splicing events were quantified using **rMATS** (v4.3.0) with paired-end mode enabled, applying both junction counts and exon body counts (JCEC mode). rMATS computed percent-spliced-in (PSI) scores for five canonical splicing event types: skipped exons (SE), alternative 5′ splice sites

(A5SS), alternative 3′ splice sites (A3SS), retained introns (RI), and mutually exclusive exons (MXE).

4.3. Genetic variant analysis and interpretation

SNP data were generated using GATK according to the pipeline of a previous study. Variants were called via **HaplotypeCaller** (`-stand-call-conf 20 --minimum-mapping-quality 20`) and filtered with **VariantFiltration** (`--filter-name FS -filter "FS > 30.0" --filter-name QD -filter "QD < 2.0"`). Only dbSNP-annotated variants, excluding UCSC RepeatMasker microsatellites and REDIportal database entries, were retained.

5. Build of standardized data-sharing frameworks

All data matrices were organized into SummarizedExperiment (SE) or RaggedExperiment (RE) objects, with genomic coordinates of the reference features (defined in Sections 4.1–4.3) stored in `rowRanges()` and sample-level metadata in `colData()`.

For epitranscriptome SE objects, the following matrices can be accessed through `assays()`:

- **m6A counts** and **Total counts** (coverage per reference site),
- **BBmix posterior probabilities** (foreground component responsibilities, stored as `m6ASiteProb`), and
- **BH-adjusted p-values from binomial tests**.

For multi-omics integration, MultiAssayExperiment (MAE) was used to combine assay-specific objects. Sample identifiers were harmonized across assays using curated metadata tables to ensure consistent mapping of samples between techniques.

Step-by-step instructions for retrieving counts, probabilities, p-values, and metadata from SE/RE/MAE objects are provided in the m6AConquer Data Framework Usage Tutorial.

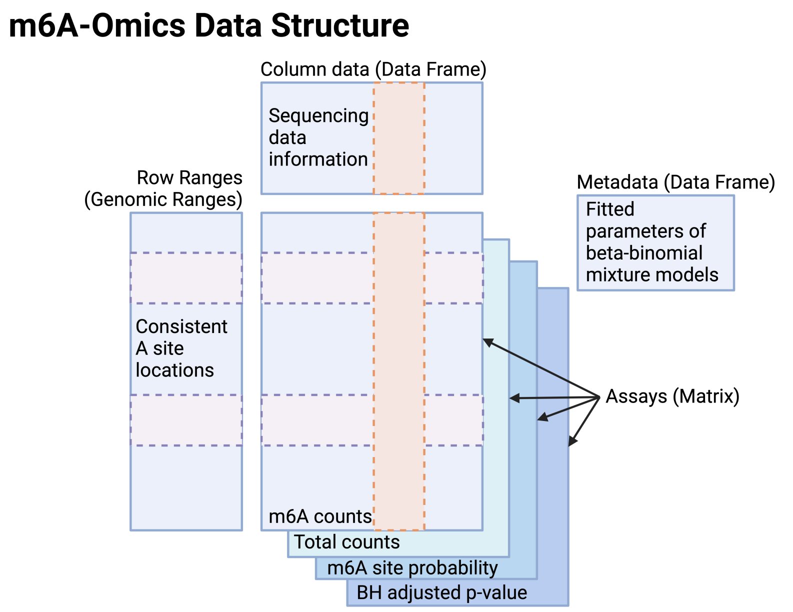

Data structure of SummarizedExperiment (SE) objects; using m6A methylome matrices as an example.

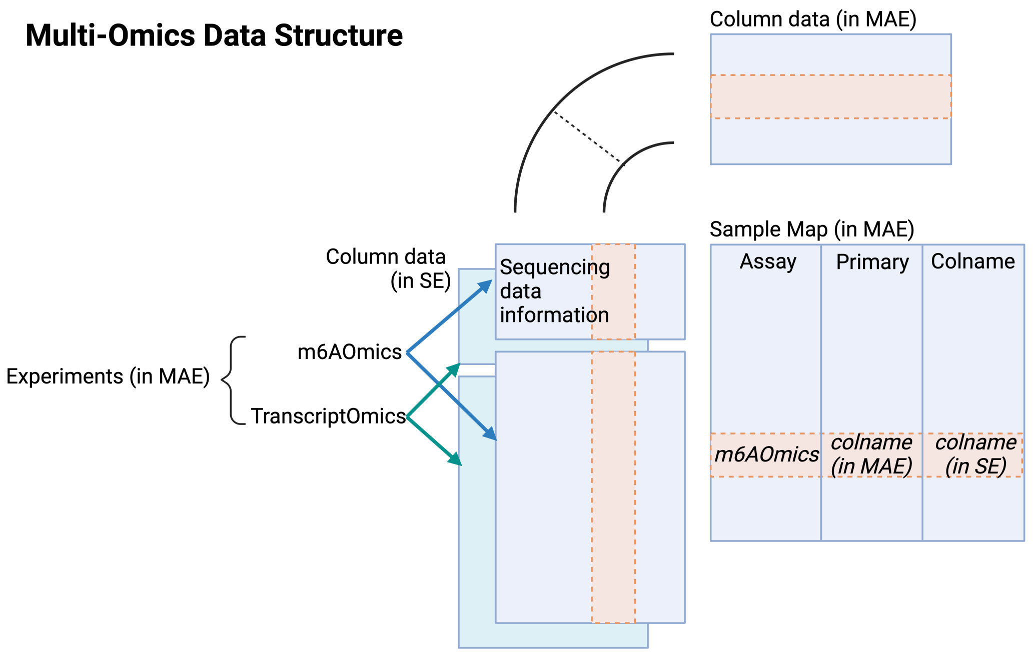

Data structure of Multi-Assay Experiment (MAE) objects; Using m6A methylome and transcriptome as examples.

6. m6A QTL analysis and disease association

A total of 211 MeRIP-seq samples under baseline wild-type conditions (no knockdown/knockout or treatment) were analyzed for m6A-QTL mapping. Raw methylation levels at human orthogonally validated sites were quantified as logit-transformed methylation proportions (*log((IP * sum(input))/(input * sum(IP)))*). Note

that the counts were normalized by sequencing depth of the paired IP and input libraries.

To ensure data quality, sites (matrix rows) with >20% missing values (defined as loci with zero coverage in both IP and input libraries) across samples were removed first. Then, samples (matrix columns) with >80% missing loci (zero total coverage) were also excluded.

Next, a linear model was applied per sample to adjust for variation in IP efficiency, with global shifts normalized relative to a reference sample (the site wised average across all samples). GC-content bias was then removed by fitting natural cubic spline–smoothed linear models, where GC content was computed from a 201 bp symmetric exonic window (100 bp upstream + site + 100 bp downstream).

The corrected M-levels were then z-standardised per site across samples and quantile-normalised per sample across sites to harmonize value distributions. Latent factors were estimated using singular value decomposition (SVD), with the number of factors determined via the R function `num.sv` from the SVA package. In this dataset, 47 latent factors were identified. Finally, these latent factors, together with known batch and condition covariates, were incorporated as the covariate design matrix for m6A QTL mapping.

Cis-m6A QTL mapping was performed using FastQTL v2.184 (118). Two empirical stringency thresholds were applied to identify target m6A sites: empirical $p < 0.1$ (low) and < 0.05 (high). Empirical p-values were derived from 10,00 permutations per site, with beta approximation enabled (`--permute 1000 --beta`). Candidate cis-SNPs were restricted to gene bodies (exons and introns defined by Ensembl v110) and promoter regions (−2000 bp upstream to +200 bp downstream of the transcription start site). For each m6A site, nominal p-value thresholds corresponding to the two empirical thresholds were obtained directly from the FastQTL permutation output (`thresholds.txt`).

Disease relevance was assessed by annotating m6A-QTL variants with GWAS Catalog and ClinVar datasets, following the annotation framework of a previous study. Variants were annotated using **VEP v107** with dbSNP build 155, and GWAS/ClinVar matches were identified by rsID. To extend associations through linkage disequilibrium (LD), we used PLINK v1.9.0 with the 1000 Genome Project Phase 3 (hg38) reference panel, applying `--r2 --ld-window-kb 1000 --ld-window 99999 --ld-window-r2 0.8`. The resulting LD-expanded variant set was then mapped separately to GWAS-associated traits and ClinVar-curated phenotypes, producing two independent disease association tables.
