# Supplementary material for: m6AConquer: a consistently quantified and orthogonally validated database for the N6-methyladenosine (m6A) epitranscriptome
Source: Nucleic Acids Res. 2025 Dec 3;54(D1):D204–18. doi: 10.1093/nar/gkaf1204 (PMC12807759; doi:10.1093/nar/gkaf1204)
Supplement: gkaf1204_Supplemental_Files [file gkaf1204_supplemental_files.zip › Supplementary-Figures.pdf]

Supplementary Figures

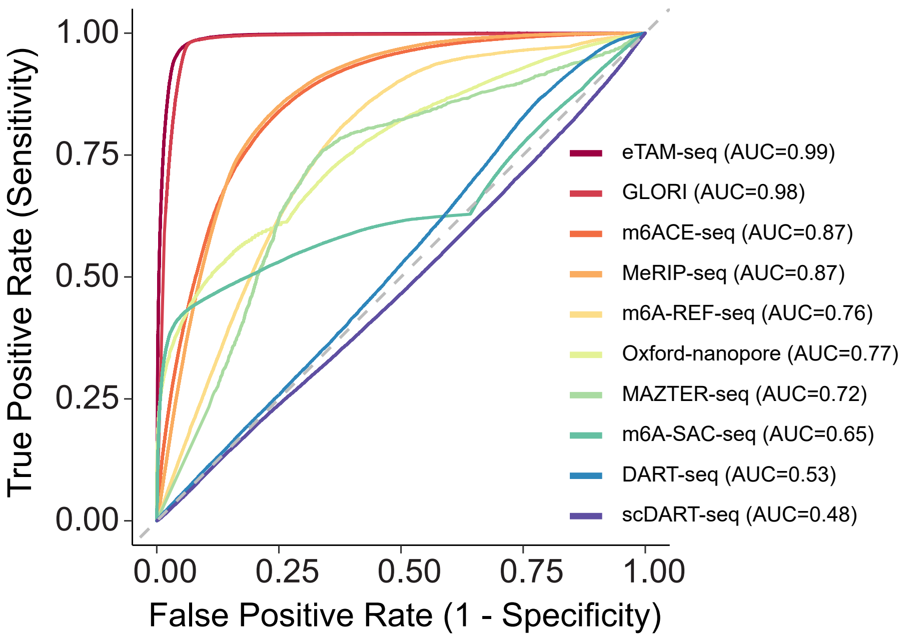

Supplementary Figure S1: ROC curves evaluating the m^{6}A profiling technique accuracy using orthogonally validated sites as ground truth. The posterior probability of m6A foreground returned by BBmix was used as the predictive value.

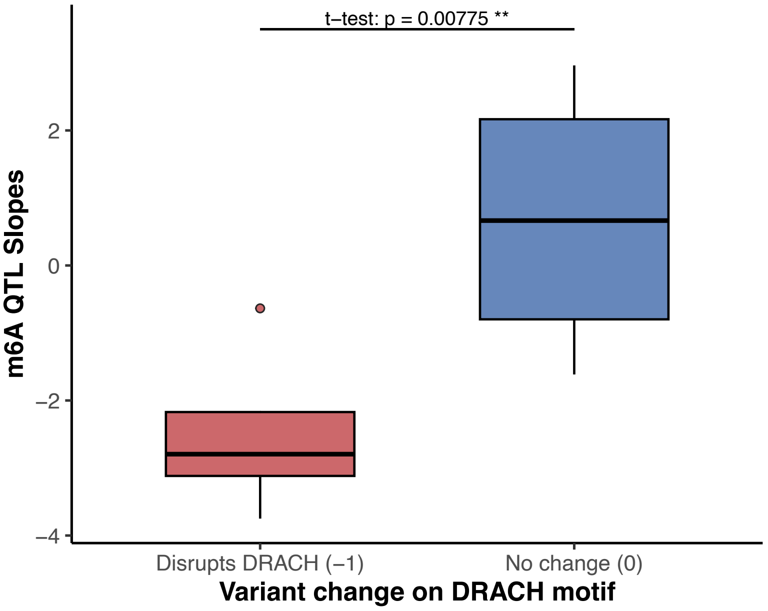

Supplementary Figure S2: To assess the quality of our m6A QTL dataset, we selected m6A QTLs (under empirical p-value < 0.1) whose associated variants lie within the DRACH motif of the corresponding m6A sites. We observed that variants disrupting the DRACH motif have significantly more negative m6A QTL effect sizes compared to those that do not (t-test p = 0.00775). It is worth noting that, in our dataset, all observed slopes for DRACH-disrupting variants were negative.

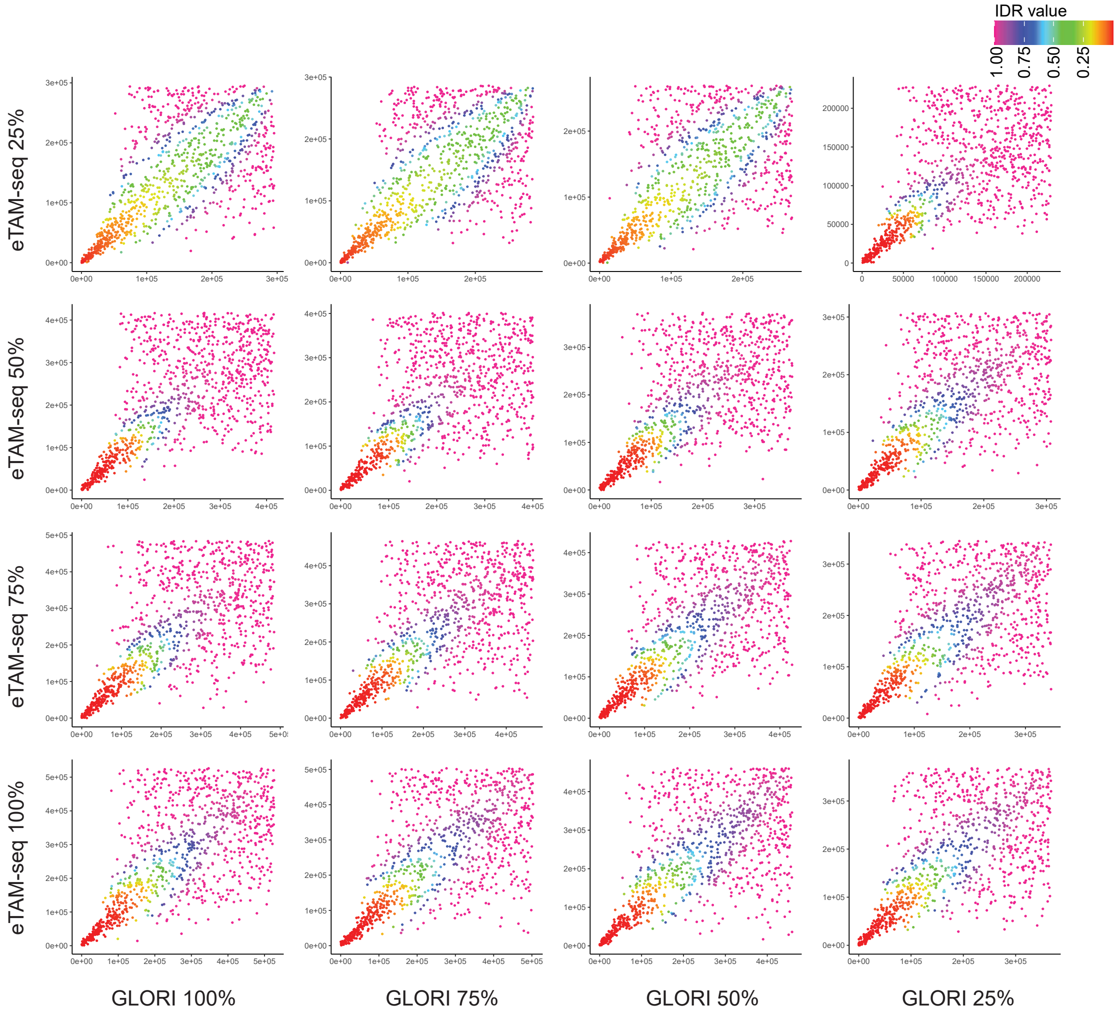

Supplementary Figure S3: To investigate the robustness of our IDR integration strategy with respect to sequencing depth, we performed a series of read down-sampling experiments (with replacement) on the combined GLORI and eTAM-seq datasets. The resampling/bootstrap was simulated using the *rhyper* function in R. Across down-sampling levels of 75%, 50%, and 25%, the IDR integration outcomes, such as the reproducible fractions, remained relatively unchanged. This result is expected, as our IDR strategy requires coverage ≥ 20 in both techniques, which stabilizes the beta-value estimates regardless of sequencing depth.

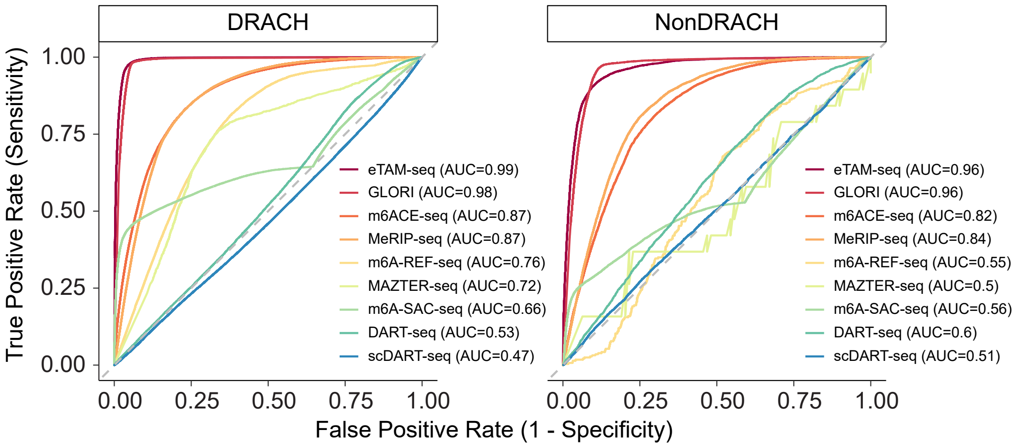

Supplementary Figure S4: In order to rule out potential biases in our definition of reference sites, we performed the same AUROC evaluation as in Supplementary Figure 1, but stratified by DRACH motif sites (mostly from exon references) and non-DRACH motif sites (derived exclusively from GLORI data). The results show that the relative performance ranking of different methods remained largely unchanged, with eTAM-seq and GLORI still achieving the highest predictive performance on our validated sites. This suggests that selection bias from GLORI reference sites is relatively weak, and most non-motif sites supported by GLORI can also be detected by other techniques.
